# Supplementary material for: Probing relaxed myosin states in hypertrophic cardiomyopathy by second harmonic-generation microscopy
Source: eLife. 2026 Aug 17;14:RP107730. doi: 10.7554/eLife.107730 (PMC13480991; doi:10.7554/eLife.107730)
Supplement: Peer review history

# Editor report: eLife Assessment

**DOI:** 10.7554/eLife.107730.3.sa0

**Authors:** James R Sellers

**Affiliations:** National Institutes of Health United States

**Keywords:** Convincing, Fundamental

This **fundamental** study demonstrates that polarized second-harmonic generation microscopy can be used to probe the ON/OFF states of myosin in both permeabilized and intact muscle, making this key measurement accessible to a greater number of labs. This has the potential to help with the study of disease-causing mutations and our understanding of drug function. The methodology is well defined, and the results are **convincing**; however, there are some limitations to the interpretation of the data.

# Referee report: Reviewer #2 (Public review)

**DOI:** 10.7554/eLife.107730.3.sa1

Summary:

In striated muscle, myosin motors can dynamically switch between an energy-conserving OFF state and an activated-ON state. This switching is important for meeting the body's needs under different physiological conditions, and previous studies have shown that disease causing mutations associated with cardiomyopathies can affect the population of these states, leading to aberrant contractility. Studying these structural states in muscle has previously only been possible via X-ray diffraction which requires access to a beam line. Here, Arecchi et al. demonstrate that polarized second-harmonic generation microscopy (pSGH), a technique that is more accessible, can be used to probe the ON/OFF states of myosin in both permeabilized and intact muscle.

Comments on revised version:

The manuscript has been significantly strengthened in the revision. The authors have addressed my concerns.

# Referee report: Reviewer #3 (Public review)

**DOI:** 10.7554/eLife.107730.3.sa2

Summary:

This is a very interesting paper extending the use of SHG to the study of relaxed muscle and its use to assess the order- disorder (and on /off) states of myosin heads in the thick filament. The work convincingly shows that SHG, and the parameter gamma, provide a reliable measure of the state of the myosin heads in a range of different relaxed muscle fibres, both intact and skinned and in myofibrils. In mini pig cardiac fibres the use of dATP and mavacamten increased or decreased the number of heads in the disordered state respectively. On the assumption that these treatments push myosins fully into the disordered or ordered state then this allows the fraction of ordered heads to be assessed under a wide variety of conditions. The extension of this part of the study to mouse heart and rabbit psoas samples extends the validation of the approach.

The results with the myosin mutant R403Q support the idea that this mutation reduces the fraction of myosin heads in the ordered state and that mavacamten can recover the WT situation.

The results from SHG were compared with parallel studies using X-rays to validate the conclusions. Independent fibre ATPase data further support the conclusions.

The work is solid and provides a novel approach assessing the activity state of muscle thick filaments. The authors point out some of the potential uses of this approach in the future including time resolved SHG measurements. Indeed, jumps in mavacamten or dATP concentration with time resolved SHG could measure the rates of entry and exit from the ordered , off state of the filament. A measurement urgently needed in the field.

Strengths:

(1) The SHG signal is convincingly shown to assess the fraction of ordered/disordered myosin heads in the thick filament of a variety of muscle fibres.

(2) The results are similar for rabbit psoas, mouse and minipig cardiac fibres, Skinning the fibres and production of myofibrils does not change the SHG signal.

(3) Use of myosin R403Q mutant in mini pig confirms a loss of ordered myosin heads and the ordered heads can be recovered by mavacamten.

(4) Parallel X-ray scattering and ATPase data support the conclusions.

(5) Assuming that dATP and mavacamten generate 100% disordered vs ordered myosin heads respectively then the % ordered heads can be calculated for a variety of conditions.

(6) The potential of extending the technique with time resolved studies and sub sarcomere variations of SHG are very exciting prospects.

Weaknesses:

Issues like the effect of fibre disarray on the SHG signal are not well defined.

# Author comment: Author response

**DOI:** 10.7554/eLife.107730.3.sa3

**Authors:** Giulia Arecchi, Marica Dente, Weikang Ma, Beatrice Scellini, Nicoletta Piroddi, Marina Scardigli, Jingyuan Yu, Jing Zhao, Riccardo Cicchi, Ryo Kinegawa, Caroline Muellenbroich, Francesco Sera, Corrado Poggesi, Cecilia Ferrantini, Thomas Irving, Michael Regnier, Leonardo Sacconi, Chiara Tesi

**Affiliations:** University of Florence Florence Italy; University of Florence Florence Italy; Illinois Institute of Technology Chicago, IL United States; University of Florence Florence Italy; University of Florence Florence Italy; University of Florence Florence Italy; Dalian Medical University Dalian China; Dalian Medical University Dalian China; National Institute of Optics Florence Italy; University of Glasgow Glasgow China; University of Glasgow Glasgow United Kingdom; University of Florence Florence Italy; University of Florence Florence Italy; University of Florence Florence Italy; Illinois Institute of Technology Chicago United States; University of Washington Seattle United States; Istituto di Fisiologia Clinica Florence Italy; University of Florence Florence Italy

The following is the authors’ response to the original reviews.

**Public Reviews:**

**Reviewer #1 (Public review):**

This study utilizes polarized second-harmonic generation (pSHG) microscopy to investigate myosin conformation in the relaxed state, distinguishing between the disordered, actin-accessible ON state and the ordered, energy-conserving OFF state. By pharmacologically modulating the ON/OFF equilibrium with a myosin activator (2deoxyATP) and inhibitor (Mavacamten), the authors demonstrate that pSHG can sensitively quantify the ON/OFF ratio in both skeletal and cardiac muscle. Validation with X-ray diffraction supports the accuracy of the method. Applying this approach to a hypertrophic cardiomyopathy model, the study shows that R403Q/MYH7-mutated minipigs exhibit an increased ON state fraction relative to controls. This difference is eliminated under saturating concentrations of myosin modulators, indicating that the ON/OFF balance can be pharmacologically shifted to its extremes. Additionally, ATPase assays reveal elevated resting ATPase activity in R403Q samples, which persists even when the ON state is saturated, suggesting that increased energy consumption in this mutation is driven by both a shift toward the ON state and inherently higher myosin ATPase activity.

Strengths:

This is a well-written and well-conducted study that clearly reveals the power of SHG microscopy. The study clearly establishes the great utility of SHG to study thick filament regulation.

Weaknesses:

(1) Several studies have shown that the ON state of the thick filament is sensitive to both temperature and filament lattice spacing, with a common recommendation to conduct skinned fiber experiments at temperatures above 27 °C and in the presence of dextran to better preserve physiological conditions. The authors should clarify the experimental temperature used in their skinned fiber studies, indicate whether dextran was included, and discuss whether adherence to these recommended conditions would have impacted their results.

Additional experiments were performed on skinned psoas muscle strips to assess the influence of temperature and lattice spacing on the measured γ values. In particular, psoas strips were tested under relaxing conditions at three different temperatures (10 °C, 15 °C, and 20 °C). In a separate set of experiments, psoas strips were examined under relaxing conditions in the absence and in the presence of 5% dextran.

These measurements confirmed previous reports indicating that the ON state of the thick filament is sensitive to both temperature and lattice compression. Specifically, increasing the temperature from 10 °C to 20 °C resulted in a shift toward lower γ values, consistent with a greater radial displacement of myosin heads. A similar trend, although less pronounced, was observed in the presence of dextran, which also produced a decrease in γ compared with control conditions.

Figure 1 and text have been modified including these new findings (revised manuscript, pages 11-12).

(2) On page 13, the authors report the proportion of disordered heads as approximately 30% in wild-type and 65% in R403Q fibers. They should clarify whether these values represent the percentage of total myosin heads, or rather the percentage of heads that are responsive to Mavacamten and dATP.

The proportions reported in the original version referred to the fraction of myosin heads assumed to be responsive to Mavacamten and dATP. These estimates were obtained using a simplified model in which dATP was assumed to produce a complete (100%) shift of myosin heads toward the ON state, whereas Mavacamten was assumed to cause a complete depletion (0%).

Since these assumptions are not fully supported by structural data, we decided to remove these values from the Results section and instead discuss these considerations in more general terms in the Discussion (page 19). Figure 3 and text have been modified accordingly (pages 14-15).

(3) In Figure 5, regarding ATPase measurements, the content of contractile material per unit volume of muscle preparation will influence the results. Did the authors account for this variable, and if not, how might it have affected the conclusions?

We thank the reviewers for raising this concern and agree that the content of contractile material per unit volume can influence the absolute values obtained in ATPase measurements. In our experiments, however, we primarily assessed the effects of compounds using paired measurements (e.g., the same strip measured before and after Mavacamten treatment).

In addition, based on our previous structural data obtained from similar preparations of human myocardium (https://doi.org/10.1161/CIRCRESAHA.122.321956), the ratio of contractile tissue volume to total muscle volume is highly preserved. This supports comparisons between muscle strips from different experimental groups (e.g., WT and R403Q).

(4) For readers primarily interested in assessing the ON/OFF state of thick filaments, could the authors list the specific advantages of polarized second harmonic generation (pSHG) microscopy compared to X-ray diffraction?

In the present work, we deliberately chose to frame pSHG microscopy and X-ray diffraction as complementary approaches, rather than emphasizing a direct one-to-one comparison of their respective advantages and disadvantages.

That said, we agree that for readers specifically interested in assessing the ON/OFF state of thick filaments, a clearer delineation of the respective strengths and limitations is useful. We have therefore slightly revised the Discussion (page 21) to better clarify the advantages and constraints of both approaches.

(5) Given that many data points were derived from the same fiber or myocyte, how did the authors address the risk of type I errors due to non-independence of measurements? Was a nested or hierarchical statistical approach used?

We thank the reviewer for raising this important point and agree that the original statistical analysis did not fully account for the non-independence of measurements derived from the same fibre.

To address this issue, we have now reanalyzed the entire dataset with the support of Prof. Francesco Sera, who has been included as a co-author. A hierarchical (mixed-effects) statistical model was applied, explicitly accounting for the nested structure of the data, repeated measurements, and unbalanced group sizes.

Importantly, this revised analysis substantially confirms the original results, with no major changes in the observed trends or in the statistical significance of the findings. All figures have been modified accordingly, and the statistical methods used are now described in the Methods section (page 8).

**Reviewer #2 (Public review):**

Summary:

In striated muscle, myosin motors can dynamically switch between an energyconserving OFF state and an activated ON state. This switching is important for meeting the body's needs under different physiological conditions, and previous studies have shown that disease-causing mutations associated with cardiomyopathies can affect the population of these states, leading to aberrant contractility. Studying these structural states in muscle has previously only been possible via X-ray diffraction, which requires access to a beam line. Here, Arecchi et al. demonstrate that polarized second-harmonic generation microscopy (pSGH), a technique that is more accessible, can be used to probe the ON/OFF states of myosin in both permeabilized and intact muscle.

Strengths:

(1) There is an outstanding need in the field to better understand the regulation of the ON/OFF states of myosin. Currently, this is studied using X-ray diffraction, meaning that it is accessible to only a few labs. The authors demonstrate that pSGH can be used to probe the ON/OFF states of myosin both in intact and permeabilized muscle. This is a significant advance, since it makes it possible to study these states in a standard research laboratory.

(2) The authors demonstrate that this approach can be employed in both skeletal and cardiac muscle. Importantly, it works with both porcine and mouse cardiac muscle, which are two of the most important animal models for preclinical studies.

(3) The authors manipulate the ON/OFF equilibrium using both drugs and a genetic model of hypertrophic cardiomyopathy that has been shown to modulate the ON/OFF equilibrium. Their results generally agree with previous studies conducted using X-ray diffraction as well as biochemical measurements of myosin autoinhibition.

Weaknesses:

(1) While the application of pSGH to the ON/OFF equilibrium is an important advance, there are limited new biological insights since the perturbations used here have been extensively characterized in previous studies.

We acknowledge that the biological insights provided in this study largely confirm previous findings reported in the literature. However, the primary aim of our work was to demonstrate the applicability and robustness of pSHG in probing the ON/OFF equilibrium of thick filaments. In this context, obtaining results that are consistent with established knowledge represents an important validation of the technique.

We therefore believe that the combination of these findings with the advantages of pSHG makes our work noteworthy and supports the broader utility of this approach in future biological and physiological studies.

(2) SGH has previously been applied to study the nucleotide-dependent orientation of myosin motors in the sarcomere (PMID: 20385845). The authors have previously interpreted the value of gamma as being a readout of lever arm position, but here, it is interpreted as a measure of ON/OFF equilibrium. When this technique is applied to intact muscle, it is not clear how to deconvolve the contributions of lever arm angle from the ON/OFF population (especially where there is a mix of states that give rise to the gamma value). This is an important limitation that is not discussed in the manuscript.

We thank the reviewer for this insightful and important observation. The SHG signal arises from the coherent contribution of peptide bonds within the myosin molecule and therefore reflects an average angular distribution rather than a single structural state. In the study cited (PMID: 20385845), by reconstructing each contributing second-harmonic emitter within the actomyosin motor array at the atomic scale, we were able to establish a direct relationship between myosin conformation and the γ value. In particular, this analysis revealed an overall trend: γ increases as the average angle of myosin relative to the thick filament backbone becomes larger, and decreases as this angle is reduced. Based on these observations, we proposed that γ could serve as a proxy sensitive to changes in the ON/OFF equilibrium.

However, we fully acknowledge that, especially in intact muscle, it is not possible to disentangle the respective contributions of lever arm orientation and population shifts between different structural states. The reviewer’s point is therefore well taken.

To address this, we have revised the Discussion (pages 18-19) to more clearly acknowledge this limitation and to better articulate the interpretative framework underlying our analysis.

Finally, we would like to emphasize that, in the present study, we aimed to minimize the contribution of actomyosin-bound states in intact trabeculae by performing experiments under conditions expected to strongly favor relaxation (low stimulation frequency, 0.1 Hz, and low temperature, 21 °C). Under these conditions, the number of strongly bound actomyosin cross-bridges during the diastolic phase is expected to be minimal, and variations in γ are therefore primarily associated with changes in the relaxed myosin population.

(3) The R403Q mutation has previously been shown to cause an increase in ATP usage. Here, the authors measure an elevated basal ATPase rate under relaxing conditions, and they interpret this as showing increased myosin ATPase activity intrinsic to the motors; however, care should be used in interpreting these results. Work from the Spudich lab has shown that the R403Q mutation can appear as increasing motor function in some assays but depressing motor function in others (see PMID: 32284968, 26601291). Moreover, the actin-activated ATPase rate is an order of magnitude higher than the basal ATPase rate, and thus, small changes in the basal ATPase rate are unlikely to be important for physiology.

We thank the reviewer for this thoughtful comment, and for pointing out the complexity of interpreting the functional consequences of the R403Q mutation. We agree that the functional effects of the R403Q mutation on myosin motor activity have been reported to vary depending on the experimental system and assay used, with studies showing both reduced and enhanced motor performance (PMID: 32284968, 26601291, 20560002).

Consistent with this complexity, previous biochemical and physiological studies have reported altered energetic properties associated with the R403Q mutation in cardiac muscle. In a previous study, the relationship between cross-bridge kinetics and energetics was investigated in single cardiac myofibrils and multicellular cardiac muscle strips from human HCM samples with and without the R403Q mutation. In those experiments, cross-bridge relaxation was faster in R403Q samples and correlated with an increased energetic cost of tension generation. Basal ATPase activity measured in human samples was also elevated (4.4 ± 0.5 vs 6.6 ± 1.2 μmol L^-1^ s^-1^ in HCMsn and R403Q, respectively), supporting the idea that the mutation affects energetic balance in the relaxed state (PMID: 24928957).

We also agree that actin-activated ATPase activity is substantially higher than basal ATPase activity. However, cardiac muscle spends a large fraction of the cardiac cycle in the relaxed (diastolic) state, during which myosin heads are predominantly detached from actin. Even relatively small changes in ATP turnover during this phase could therefore influence the overall myocardial energetics, and potentially contribute to the activation of signaling pathways involved in pathological remodelling.

To address the reviewer’s concern, we have now expanded the Discussion (pages 19-20) to clarify the heterogeneous results reported in the literature for the R403Q mutation, and to more cautiously interpret the physiological implications of the observed resting ATPase activity.

(4) The authors interpret some of their data based on the assumption that the high concentrations of drugs cause the myosin to either adopt 100% OFF or ON states. This assumption is not validated, limiting the ability to interpret the fraction of myosins in the ON/OFF states.

We fully agree: these assumptions are not fully supported by structural data. We consistently decided to remove these analysis from the Results section and instead discuss these considerations in more general terms in the Discussion (page 18). Figure 3 and text have been modified accordingly (pages 14-15).

(5) The ATPase measurements are innovative but hard to interpret. dATP and ATP do not have identical ATPase kinetics, meaning that it is hard to deconvolve whether the elevated ATPase rate with dATP is due to changes in the ON/OFF population and/or intrinsic ATPase activity. Similarly, mavacamten reduces the rate of phosphate release from myosin, and this effect is not strictly coupled to the formation of the OFF state (e.g., see PMID: 40118457). As such, it is difficult to deconvolve drug-based changes in the inherent ATPase kinetics of the myosin from changes in the OFF-state population.

We thank the reviewer for this important comment. We recognize that dATP and ATP do not exhibit identical ATPase kinetics, and that Mavacamten slows steps in myosin nucleotide release that are well documented for ATP and not for dATP (for both S1; PMID: 28808052 and, more markedly, HMM; PMID: 30018063). At the same time, both compounds have been shown to perturb the regulatory state of myosin heads along the thick filament. These effects are, in turn, mediated by shifts in the distribution of ATPase intermediate states, which alter the likelihood of myosin adopting autoinhibited conformations and thereby biasing the system toward OFF or ON states (see, e.g., PMID: 39444161; PMID: 30018063). This configures a dual mechanism of action for small molecules, which we now describe more clearly in the Discussion (page 20). As suggested by the referee, we also highlight the intrinsic difficulty of disentangling compound-induced changes in the intrinsic ATPase kinetics of myosin from shifts in the population of the OFF state. Consequently, we have now better focused results and discussion section considering mainly differential effects of the drugs on SHG and ATPase measurements.

Moreover, under the strongly relaxing conditions used in our experiments (pCa 10), the population of actomyosin-bound cross-bridges is expected to be extremely small (<0.1%), thereby minimizing any dATP-mediated activation of myosin arising from enhanced electrostatic interactions with actin (see, e.g., PMID: 31110001). This is supported by the absence of a significant effect of the nucleotide on the resting tension of myofibrils (new Fig. S1).

We have now better clarified these points in both result and discussion section of the revised manuscript.

**Reviewer #3 (Public review):**

This is a very interesting paper extending the use of SHG to the study of relaxed muscle and its use to assess the order-disorder (and on /off) states of myosin heads in the thick filament. The work convincingly shows that SHG and the parameter gamma provide a reliable measure of the state of the myosin heads in a range of different relaxed muscle fibres, both intact and skinned, and in myofibrils. In mini pig cardiac fibres, the use of dATP and mavacamten increased or decreased the number of heads in the disordered state, respectively. On the assumption that these treatments push myosins fully into the disordered or ordered state, then this allows the fraction of ordered heads to be assessed under a wide variety of conditions. It is unfortunate that dATP treatment was not used (as mavacmten was) on rabbit psoas and mouse samples to further test this hypothesis.

The results with the myosin mutant R403Q support the idea that this mutation reduces the fraction of myosin heads in the ordered state and that mavacamten can recover the WT situation.

The results from SHG were compared with parallel studies using X-rays to validate the conclusions. Independent fibre ATPase data further support the conclusions.

The work is solid and provides a novel approach to assessing the activity state of muscle thick filaments. The authors point out some of the potential uses of this approach in the future, including time-resolved SHG measurements. Indeed, jumps in mavacamten or dATP concentration with time-resolved SHG could measure the rates of entry and exit from the ordered, off state of the filament. A measurement is urgently needed in the field.

Strengths:

(1) The SHG signal is convincingly shown to assess the fraction of ordered/disordered myosin heads in the thick filament of a variety of muscle fibres.

(2) The results are similar for rabbit psoas, mouse, and minipig cardiac fibres. Skinning the fibres and production of myofibrils do not change the SHG signal.

(3) Use of myosin R403Q mutant in mini pig confirms a loss of ordered myosin heads, and the ordered heads can be recovered by mavacamten.

(4) Parallel X-ray scattering and ATPase data support the conclusions.

(5) Assuming that dATP and mavacamten generate 100% disordered vs ordered myosin heads respectively, then the percentage of ordered heads can be calculated for a variety of conditions.

Weaknesses:

(1) Issues like the effect of fibre disarray and lattice spacing on the SHG signal are not well defined.

We thank the reviewer for raising this important point. Regarding fibre disarray, we took advantage of the spatial resolution of SHG imaging in thick samples to selectively analyse regions of the preparation in which myofibrillar organization was preserved. This approach proved particularly useful in samples such as those from HCM, where a pronounced global disarray is present but locally well-organized regions can still be identified and reliably analysed.

Concerning lattice spacing, we have performed additional experiments on psoas muscle in which lattice spacing was modulated using dextran. This allowed us to directly assess the sensitivity of the technique to changes in inter-filament spacing.

Figure 1 and the corresponding text have been revised accordingly to incorporate and clarify this point (page 11-12).

(2) The, now well-defined heterogeneity of thick filament structure is not acknowledged.

We agree that the heterogeneity of thick filament structure is an important aspect that should be acknowledged. In the revised manuscript, we have now explicitly addressed this point in the Discussion (page 21). In particular, we highlight that the capability of pSHG to probe the ON/OFF state with sub-sarcomere spatial resolution offers the future opportunity to investigate spatial heterogeneity in thick filament organization. This aspect is now clearly acknowledged and discussed in the context of the potential applications of the technique.

(3) dATP was only used on minipig cardiac fibres. The effect of dATP on rabbit psoas and mouse cardiac fibres would be a useful comparison and would help validate the calculation of % ordered heads.

We agree that, in the original version of the manuscript, there was a methodological imbalance in the use of dATP across preparations. To address this point, we have performed additional experiments in which the effect of dATP was also evaluated in rabbit psoas and mouse cardiac fibres.

Importantly, the inclusion of these data has also proven useful in the discussion of the ON/OFF equilibrium across different muscle types and species. The corresponding results and discussion have been added to the revised manuscript (page 18-19).

**Recommendations for the authors:**

**Reviewer #2 (Recommendations for the authors):**

In addition to addressing the points in the Public Review, please also address the following points.

(1) There are some issues with the calculated ionic strengths of the solution (or some details are missing). For example, on p. 4, it is stated that there is a 200 mM ionic strength solution that contains both 100 mM KCl and 2 mM MgCl2, meaning that the ionic strength is higher than 200 mM. There are other similar issues in other places.

We thank the reviewer for pointing this out. We agree that there was an error in the reported ionic strength calculations and that some details were not sufficiently clear.

We have now corrected the ionic strength values throughout the manuscript and revised the Methods section to provide a clearer and more consistent description of the solution composition (pages 6-7).

(2) Please report standard deviations rather than standard errors.

We thank the reviewer for this suggestion. Following the recommendations of Reviewer 1, we have reanalyzed the entire dataset using an updated statistical framework. As part of this process, we carefully evaluated the most appropriate measure of variability and have now consistently reported the corresponding error estimator throughout the manuscript.

(3) For the statistical testing, please mention what tests were done for normalcy. It appears that some data is not normally distributed, in which case nonparametric tests should be used.

We have now reanalysed the entire dataset with the support of Prof. Francesco Sera, who has been included as a co-author. A hierarchical (mixed-effects) statistical model was applied, explicitly accounting for the nested structure of the data, repeated measurements, and unbalanced group sizes. As part of this updated statistical framework, normality tests were performed for all datasets. When the assumption of normality was not met, appropriate nonparametric or model-based approaches were used.

(4) Please discuss sex as a biological variable.

Sex as a biological variable was not specifically investigated in the present study, and we agree that this represents a limitation. This point has now been explicitly acknowledged in the revised manuscript (page 5).

(5) Please add an explicit section on limitations.

We thank the reviewer for this suggestion. In the revised manuscript, the Discussion has been expanded to more clearly highlight the limitations of the technique and to better contextualize them in comparison with other approaches (page 21). We believe that integrating these aspects within the Discussion provides a more coherent and balanced presentation, and we have therefore chosen not to include a separate, dedicated limitations section.

(6) Please discuss the limitations of using saturating concentrations of the drug. For example, the sensitivity of this method to detect changes in ON/OFF equilibrium at physiological concentrations is likely lower.

The Discussion has been implemented to highlight that the sensitivity of this technique to the ON/OFF ratio could be further explored across species by employing a range of concentrations of Mavacamten and dATP, thereby better capturing physiologically relevant conditions (pages 18-19).

(7) It is stated on p. 13 that R403Q has a higher sensitivity to mava versus dATP. I'm not sure this is supported by the data. The R403Q starts at a higher percentage of ON, and thus the effect size will be larger with mava, but this doesn't imply anything about sensitivity (which implies concentration dependence).

Our statement was not intended to imply a difference in sensitivity in terms of concentration dependence, but was instead based on the statistical outcome of our measurements. Specifically, while a significant response was observed in the presence of mavacamten, no statistically significant response was detected upon dATP application in the R403Q condition.

We acknowledge that this does not constitute evidence of differential sensitivity per se, and we have revised the text accordingly by removing the concept of sensitivity to avoid potential misinterpretation.

(8) Please add a discussion of the potential contributions of RLC phosphorylation to ON/OFF regulation.

We agree with the reviewer that the potential involvement of RLC phosphorylation in ON/OFF regulation is an important aspect to consider in future work, and we have now acknowledged this point in the revised Discussion.

**Reviewer #3 (Recommendations for the authors):**

Some things that need to be clarified:

(1) The comparison of skinned and intact muscle. These appear to give an unaltered SHG gamma signal (Figure 2), but a change in lattice spacing is expected between skinned and intact fibres. There is no mention of the lattice spacing of the samples or whether this was controlled. The implications are that SHG is independent of lattice spacing and/or the fraction of ordered myosin heads is independent of lattice spacing - each of which would be a useful result.

We thank the reviewer for this important observation and agree that the role of lattice spacing is a relevant factor in the interpretation of the SHG signal. To address this point, we have performed an additional series of experiments on rabbit psoas muscle in which lattice spacing was modulated using dextran. These measurements allowed us to directly assess the sensitivity of the SHG signal to changes in interfilament spacing. We observed relatively small effects, but in the expected direction.

The lack of appreciable differences between skinned and intact preparations may therefore be explained by the limited sensitivity of the technique to detect the relatively small variations in lattice spacing associated with these conditions.

(2) In comparing the WT and mutant mini pig cardiac data, the authors note that the mutant fibre has more disarray. A comment on the effect of disarray on the SHG signal would be helpful. How much of the difference between WT and mutant could be due to this disarray? What happens if more vs less disordered areas of the fibre are compared?

Myofibrillar disarray is indeed a characteristic feature of HCM tissue and is more evident in the R403Q minipig samples compared to WT. To minimize potential polarization artefacts related to structural disorganization, the entire field of view was first examined to identify regions of interest (ROIs) where sarcomeres showed minimal local disarray. Data acquisition and analysis were restricted to these locally well-aligned regions, as described in the Methods section. Therefore, the comparison between WT and mutant samples was performed on locally well-oriented regions rather than on highly disorganized areas.

We have now clarified this point in the revised manuscript to better explain how ROI selection, restricted to locally aligned regions, minimizes the potential contribution of myofibrillar disarray to the pSHG measurements (page 14).

(3) I note in Figure S3 that there is a bigger dispersion in the minipig data than mouse or rabbit. The minipig may also show a non-normal distribution. Has this been considered?

Following the reviewer’s suggestion to include dATP measurements in additional species, we have extended the interspecies analysis and revised Figure S3 to provide a direct comparison across rabbit psoas, mouse cardiac, and minipig cardiac samples under the investigated conditions.

From this more comprehensive dataset, no substantial differences in data dispersion are apparent among the different muscle types. Moreover, as part of the updated statistical framework, normality tests were performed for all datasets

(4) A note about the heterogeneity in the regulation of thick filaments along their length should be added. There is significant evidence for a difference in regulation between the MyBP-C regions and the rest from single molecule studies (Kad lab) and interference X-ray signals (London Kings group). This is probably beyond the current resolution of the SHG, but the complexity should be acknowledged. Heterogeneity in the thick filament is also apparent from cryo-EM images of relaxed muscle and thick filaments.

This important point is now acknowledged the revised Discussion (page 21).

(5) Interpretation of the effects of mavacamten on the ATPase data ae complicated by the observation that mava is an inhibitor of myosin independent of the effect on the order- disorder of thick filaments. I.e. mavacamten will inhibit myosin S1.

This point has now been addressed (see response to Reviewer #2, point 5 weaknesses).

Minor issues:

(1) P7 line 2: where/were purchased from Sigma.

(2) P7 last but one line: R403Q/R4303Q.

(3) P8 last line: 2-deaoxyATP 2-deoxyATP.

(4) Results, p9: in the section title and first line, replace psoas with rabbit psoas.

(5) Line 5: ROI is not defined, only in the Figure legend.

(6) Mavacamten: 50 uM used in psoas and 10 uM in cardiac fibres. A note on why would help those not familiar with this literature. Similarly, in Figure S3, presumably the mava concentration was saturating in each case.

(7) P14, last paragraph, line 3: Figure 4 should be Figure 5.

(8) P17, 5 lines from the end: as effective at 2-deoxyATP at/as.

(9) P19, lt line: fibber/fiber.

All these minor points have been fully addressed. We thank the reviewer for noting them.
